# Supplementary material for: Cleavage of MEP-1 by DPF-3 Reveals Novel Substrate Specificity and Its Impact on Reproductive Fitness
Source: bioRxiv. 2025 Sep 28:2025.09.26.678732. Preprint. [Version 1] doi: 10.1101/2025.09.26.678732 (PMC12485953; doi:10.1101/2025.09.26.678732)
Supplement: 1 [file NIHPP2025.09.26.678732v1-supplement-1.pdf]

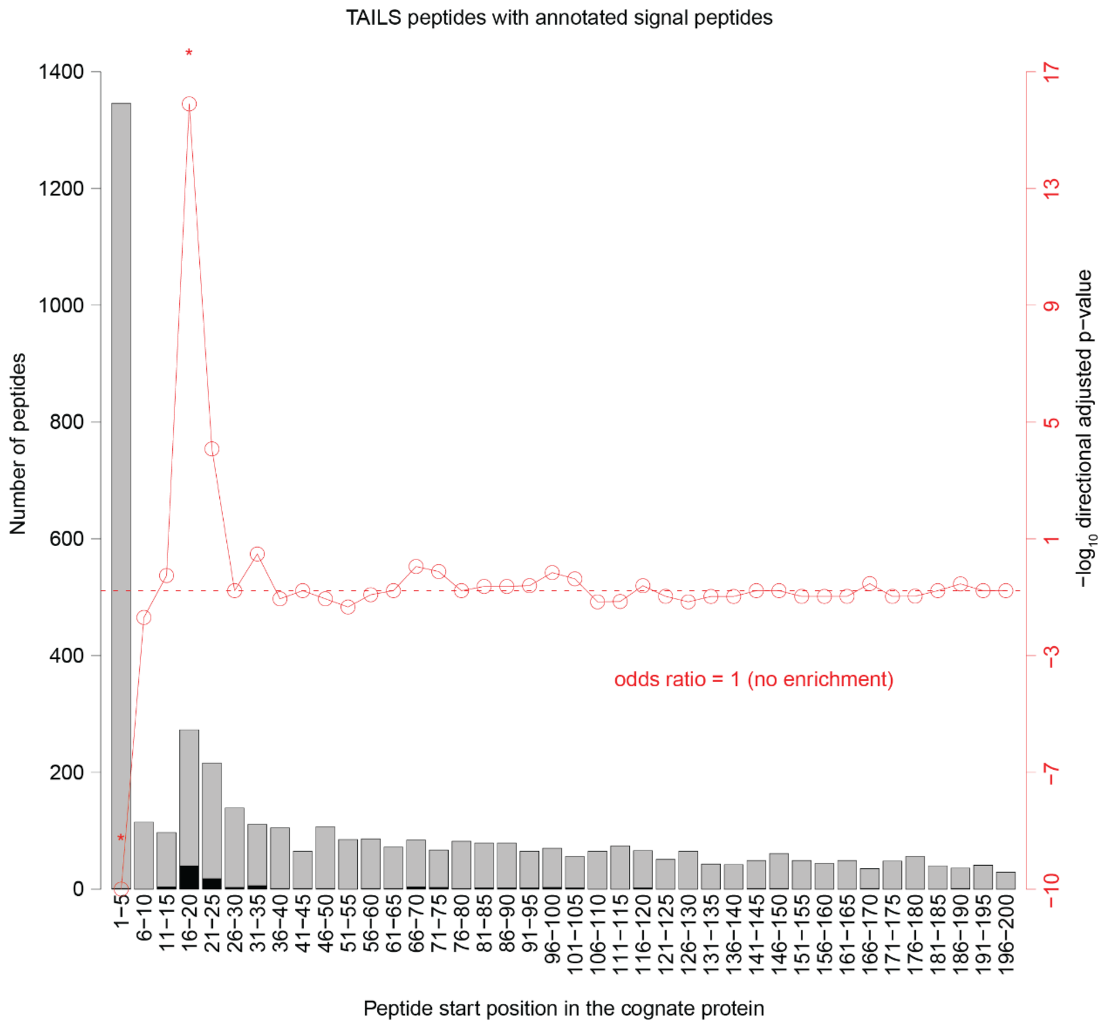

Supp. Fig. 1. TAILS enriches protein N-termini
Starting positions of peptides identified by TAILS are shown in 5 amino acid bins relative to the annotated start site for peptides originating within the first 200 amino acids. Peptides derived from proteins with an annotated signal peptide are indicated in black. The negative $\log_{10}$ directional, adjusted p-values (Fisher's exact test) testing for the enrichment of signal peptides in proteins at a given bin position are shown in red. Null hypothesis: the fraction of signal peptide annotations is independent of the start position (odds ratio = 1). There is a significant under representation of annotated signal peptides in the TAILS peptide position bins 1-5, and an over representation between 16 and 25 (red asterisks).

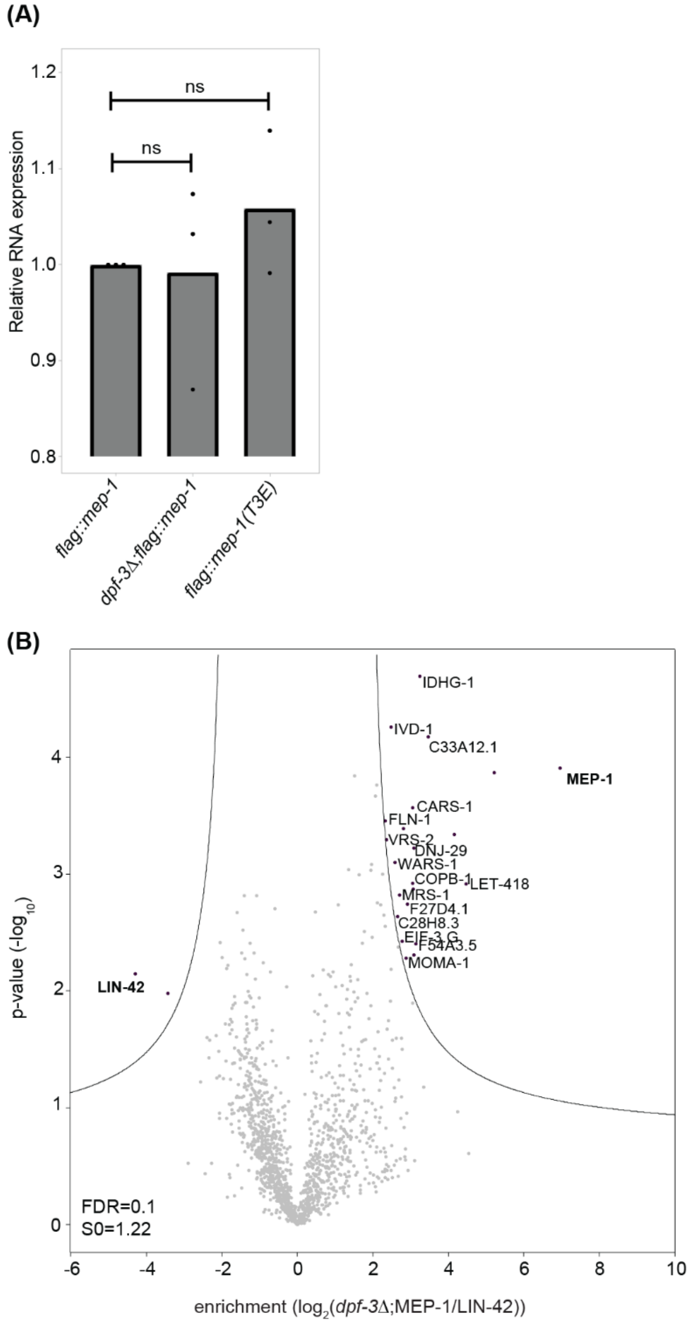

Suppl. Fig. 2. ***mep-1*** **transcript levels are unaffected by** ***dpf-3*****, and MEP-1 interactors are unchanged in** ***dpf-3Δ*** **mutant animals.**
A. RT-qPCR quantification of *mep-1* mRNA in wild-type, *dpf-3Δ*, and *mep-1(t3e)* mutant animals showing no significant differences in transcript abundance.
B. IP-MS of MEP-1. The x-axis indicates the fold enrichment of the bait protein (MEP-1 from *dpf-3Δ* animals) and its interacting partners over control (LIN-42). Each dot represents a protein, the non-significant and the significant proteins are colored gray and black, respectively. False Discovery Rate (FDR) of 0.1 was used. The y-axis of the volcano plot indicates the p-values that were calculated by two-tailed Student t-test.
